# Endometrial mesenchymal stem cells isolated from menstrual blood repaired epirubicin-induced damage to human ovarian granulosa cells by inhibiting the expression of Gadd45b in cell cycle pathway

**DOI:** 10.1186/s13287-018-1101-0

**Published:** 2019-01-03

**Authors:** Zhongrui Yan, Fengyi Guo, Qing Yuan, Yu Shao, Yedan Zhang, Huiyan Wang, Shaohua Hao, Xue Du

**Affiliations:** 1 Department of Obstetrics & Gynecology, General Hospital, Tianjin Medical University, NO.154, Anshan Road, Heping District, Tianjin, 300052 China; 2 Maternal and Child Health Care Hospital of Shandong Province, NO.238, Jingshi Road, Jinanlixia District, Jinan, 250014 China; 3 Maternal and Child Health Hospital of Guiyang City, NO.63, Ruijin south Road, Nanming District, Guiyang City, 550003 Guizhou Province China; 4 Reproductive Medical Center, Tianjin Central Hospital of Gynecology Obstetrics, NO.156, Nankai Sanma Road, Nankai District, Tianjin, 300010 China

**Keywords:** Menstrual blood, Mesenchymal stem cells, Granulosa cells, Premature ovarian failure, Epirubicin, Gadd45b, CyclinB1, CDC2

## Abstract

**Background:**

To explore the effect of mesenchymal stem cells isolated from menstrual blood (MB-MSCs) on epirubicin-induced damage to human ovarian granulosa cells (GCs) and its potential mechanisms.

**Methods:**

The estradiol, progesterone, anti-Müllerian hormone, inhibin A, and inhibin B levels were determined using enzyme-linked immunosorbent assay. The proliferation of GCs was detected by Cell Counting Kit-8 assays. The cell cycle distribution was detected by propidiumiodide single staining. The apoptosis of GCs was determined using Annexin V and 7-AAD double staining. The differentially expressed genes of GCs were analyzed with Affymetrix Human Transcriptome Array 2.0 gene chip and verified with Western blot analysis.

**Results:**

Epirubicin inhibited the secretion of estradiol, progesterone, anti-Müllerian hormone, inhibin A, and inhibin B and the proliferation of GCs; arrested these GCs in G2/M phase; and promoted the apoptosis of GCs. However, MB-MSCs repaired epirubicin-induced damage to GCs. Differentially expressed genes of GCs, Gadd45b, CyclinB1, and CDC2, were found by microarray and bioinformatics analysis. Western blot showed that epirubicin upregulated Gadd45b protein expression and downregulated CyclinB1 and CDC2 protein expression, while MB-MSCs downregulated Gadd45b protein expression and upregulated CyclinB1 and CDC2 protein expression.

**Conclusions:**

MB-MSCs repaired epirubicin-induced damage to GCs, which might be related to the inhibition of Gadd45b protein expression.

## Background

Epirubicin has become the first-line treatment for a variety of cancers, such as breast cancer, leukemia, and lymphoma, due to its broad-spectrum anticancer activity, high specificity, and low cardiac toxicity. Long-term administration of high-dose chemotherapy may cause ovarian damage in patients of fertile age, resulting in a reduction in ovarian function, amenorrhea, and finally premature ovarian failure (POF). It was reported that approximately 68% of breast cancer survivors who received chemotherapy complained of more physical and menopausal symptoms than their healthy counterparts, and some patients even had reduced ovarian reserve and reproductive potential [1]. Chemotherapy-induced POF not only has a detrimental effect on female gonadal function, but it also increases the risk of a variety of diseases such as cardiovascular diseases, osteoporosis, and cognitive disorders [2–5]. Recent studies have shown that anthracycline such as epirubicin can cause ovarian granulosa cell (GC) apoptosis and follicle damage, resulting in a reduction in ovarian function and finally the development of POF [6, 7]. Thus, attempts should be made to prevent or ameliorate chemotherapy-induced ovary damage.

Stem cell therapy has emerged as a promising treatment for repairing damaged tissues and functions [8, 9]. It has been reported that transplantation of mesenchymal stem cells (MSCs) isolated from bone marrow, fat, or umbilical cord blood can inhibit GC apoptosis, improve ovarian function, and repair ovary structure damage in mice through paracrine pathways [10–12]. Meng et al. [13] isolated a population of MSC-like cells from the menstrual blood (MB) of a healthy female. These MB-MSCs have high proliferative potential, self-renewal potential, and multiple differentiation potential, and they also have many advantages over stem cells derived from other sources, such as ease of collection, safe and noninvasive, no ethical concerns, and no autoimmune rejection [14]. In this study, MB-MSCs were isolated from three healthy female volunteers using the adherent method, and their effects on epirubicin-induced damage to human ovarian GCs as well as its potential mechanisms were investigated.

## Methods

### Isolation, culture, and identification of MB-MSCs

This study was approved by the ethics committee of Tianjin Medical University, and written informed consent was obtained from each donor. MB-MSCs were isolated and identified by adherence as previously described. Briefly, MB was collected from three healthy females aged 25–35 years, all of whom had normal menstrual cycles with no transmitted diseases such as hepatitis B, hepatitis C, syphilis, and acquired immune deficiency syndrome. Flow cytometry showed that cells isolated from MB were positive for MSC markers (CD44, CD29, CD73, and CD105), but negative for the endothelial marker CD31 and the pan-leukocyte marker CD45, respectively, indicating that these cells were MSCs rather than endothelial or hematopoietic cells, and MB-MSCs could differentiate into lipoblasts, osteoblasts, and chondroblasts [15].

### Isolation, culture, and identification of ovarian GCs

Human ovarian GCs were obtained from patients who received in vitro fertilization-embryo transfer (IVF-ET) and follicle puncture in the Reproductive Center of Tianjin Medical University General Hospital from May 2017 to February 2018. Those patients with oviduct obstruction or infertility due to male factors but normal menstrual cycles and ovulation and endocrine functions were eligible for this study. Systemic diseases were excluded. They were injected intramuscularly with 5000–10,000 U human chorionic gonadotropin (hCG), followed by transvaginal follicular aspiration 34–36 h later. Eggs were removed under the microscope, and follicular fluid was collected and centrifuged at 1500 r/min for 5 min. After that, the supernatant was removed, and cells were resuspended in PBS, transferred to the same volume of Ficoll lymphocyte separating medium (Sigma, St. Louis, MO, USA), and centrifuged at 2000 r/min for 30 min. The middle tunica albuginea layer was carefully sucked. Cells were resuspended in PBS, and the number of cells was counted using a hemocytometer. Cells were inoculated at a density of 1 × 10^6^/mL in a six-well plate at 37 °C in an atmosphere of 5% CO_2_-95% air. The culture medium was changed on the next day, and immunohistochemical (IHC) staining was performed using the streptavidin peroxidase (S-P) method 48 h later to determine the expression of follicle-stimulating hormone receptors (FSHR), which was the specific marker of GCs. The primary FSHR antibody was purchased from Bioworld Technology (St. Louis Park, MN, USA), and PV-6001 rabbit two-step IHC kits and DAB kits were purchased from ZSGB-BIO (Beijing, China), respectively.

### Effects of epirubicin on GCs in vitro

GCs were inoculated in a 96-well plate at a density of 1 × 10^4^ cells/well with the presence of 100 μl of DMEM/F12 medium containing 10% FBS, and the culture medium was replaced on the next day with 100 μl of serum-free DMEM/F12 medium containing 0, 3.125, 6.25, 12.5, 25, or 100 μg/L of epirubicin (Hisun, ZheJiang, China). Three parallel wells were set for each group and treated for 0, 6, 12, 24, 48, and 72 h, respectively, followed by culture with 10 μl of CCK8 (Beyotime, Shanghai, China) at 37 °C in an atmosphere of 5% CO_2_–95% air for 4 h. OD was measured at 450 nm using an ELISA reader (Lab system Multiskan, Finland), and the dose-effect curves were plotted and the half-inhibitory concentration was calculated.

### Effects of MB-MSCs on GCs

GCs were cultured alone (control group) or co-cultured with 25 μg/L of epirubicin (G+E group) or MB-MSCs after treatment with epirubicin (G+E+M group). Three parallel wells were set for each group. GCs were seeded at a density of 5 × 10^5^ cells/well in a six-well plate and incubated with 3 mL of DMEM/F12 medium containing 10% FBS at 37 °C in an atmosphere of 5% CO_2_-95% air. After 24 h, the supernatant was removed, and the culture medium was replaced with 3 mL of serum-free DMEM/F12 medium in the control group and 3 mL of serum-free DMEM/F12 medium containing 25 μg/L of epirubicin in the G+E and G+E+M groups, respectively. On the next day, all culture media were replaced with 3 mL of serum-free DMEM/F12 medium, and a cell culture insert (Corning, NY, USA) was added in the G+E+M group, where MB-MSCs were seeded at a density of 2 × 10^5^ cells/well in order to allow the pass of cytokines but impede the pass of MB-MSCs, and then GCs and MSCs were co-cultured in the same culture medium. After 24 h, 3 mL of supernatant and GCs was collected, respectively.

### Enzyme-linked immunosorbent assay

The E2, progesterone, AMH, inhibin A, and inhibin B levels in the supernatant were determined using an ELISA kit (Cloud-Clone Corp, TX, USA) to evaluate the functions of GCs according to the manufacturer’s protocol.

### CCK8

GCs were collected from all groups after 24, 48, and 72 h of culture, respectively. GCs were seeded at a density of 1 × 10^4^ cells/well in a 96-well plate and detected using a CCK8 kit according to the manufacturer’s protocol.

### Propidiumiodide (PI) single staining

GCs were collected from all groups after culture, washed with PBS, fixed with 70% ethanol at 4 °C overnight, and then washed again. Cells were incubated with 1 mg/mL RNase A (Solarbio, Beijing, China) at 37 °C for 30 min, and then with 50 μg/mL PI (Solarbio, Beijing, China) at 37 °C for 30–40 min. After that, cells were filtered by silk cloth and cell cycle distribution was analyzed by flow cytometry (FACSCalibur, BD, USA).

### Annexin V and 7-AAD double staining

The apoptosis rate of GCs was examined with an Annexin V and 7-AAD apoptosis kit (Damao, Tianjin, China) according to the manufacturer’s protocol.

### Microarray analysis and bioinformatics analysis

For Affymetrix® microarray profiling, the total RNA was isolated from GCs of the three groups using TRIzol reagent (Invitrogen, Carlsbad, Canada) and purified using a RNeasy Mini Kit (Qiagen, Hilden, Germany) according to the manufacturer’s protocol. The quality and amount of RNA were determined using a UV-Vis Spectrophotometer (Thermo, NanoDrop 2000, USA) at an absorbance of 260 nm. The mRNA expression profiling was detected by Affymetrix HTA2.0 (Affymetrix GeneChip®, USA) which included 67,528 gene-level probe sets. The microarray analysis was performed using Affymetrix® Expression Console Software (version 1.2.1). Raw data (CEL files) were normalized at the transcript level using the robust multi-array average method. The median summarization of transcript expressions was calculated. The gene-level data were filtered with the probe sets in the “core” meta-probe list that represented RefSeq genes. Significant pathways of differentially expressed genes were obtained on the basis of the Kyoto Encyclopedia of Genes and Genomes (KEGG) database and Biocarta and Reactome databases. The threshold of a significant pathway was defined as *P* < 0.05 by Fisher’s exact test.

### Western blot analysis

GCs were washed with pre-cooled PBS and scraped into RIPA lysis buffer (Beyotime, Shanghai, China). The total protein concentration of GCs was analyzed using the bicinchoninic acid assay (BCA; Solarbio, Beijing, China) according to the manufacturer’s instructions. Proteins collected from different groups were electrophoresed on a sodium dodecyl sulfate (SDS)–10% polyacrylamide gel and then transferred onto polyvinylidene difluoride (PVDF) membranes (Bio-Rad, CA, USA). Then, the membranes were blocked in 5% skim milk solution at room temperature for 2 h. Anti-CycinB1 antibody (Cell Signaling Technology, MA, USA), anti-CDC2 antibody (Cell Signaling Technology, MA, USA), and anti-Gadd45b antibody (Santa Cruz Biotechnology, CA, USA) were diluted in 1× PBST containing 5% skim milk and then incubated with PBST-washed membranes at 4 °C overnight. Then, the membranes were incubated with HRP-labeled secondary antibody (Beyotime, Shanghai, China) at room temperature for 2 h. After that, the membranes were visualized using Western Lightning® Plus-ECL (PerkinElmer, MA, USA) and exposed by ImageQuant LAS 4010 Control Software (GE, Boston, USA).

### Statistical analyses

All statistical analyses were performed using SPSS 20.0 software (SPSS Inc., Chicago, IL, USA). The results were expressed as mean ± SD. The differences between groups were assessed by two-tailed student *t* test or ANOVA, and regression analysis was also performed. A two-tailed *P* value of less than 0.05 was considered to be statistically significant (indicated by an asterisk in figures).

## Results

### Characterization of GCs

**Fig. 1:**
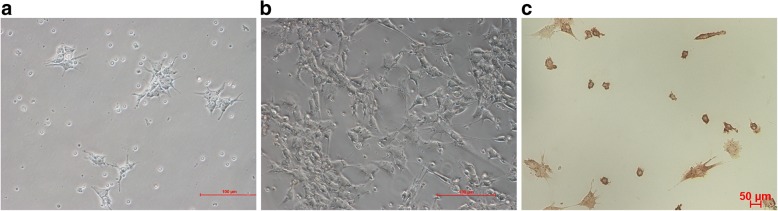
The morphology of GCs: **a** primary ovarian GCs cultured for 4 days (× 200), bar 100 μm; **b** ovarian GCs cultured for 15 days at passage 1 (× 100), bar 100 μm. **c** IHC staining of primary ovarian GCs for FSHR: both membrane and cytoplasm were brown stained (× 20), bar 100 μm

Ovarian GCs obtained from human follicular fluid were cultured for 48 h and then observed under an inverted microscope. These cells were dendritic or spindle shaped and connected by elongated pseudopodia, and they had a large and round nucleus with conspicuous nucleolus and abundant cytoplasmic particles (Fig. 1a). After the passage of GCs, cells showed a fibroblast morphology (Fig. 1b). IHC staining showed the expression of FSHR, a GC marker required for normal ovarian development and follicular maturation, in the membrane and cytoplasm. Over 90% of membranes and cytoplasm were stained brown (Fig. 1c), indicating the high purity of GCs.

### Cytotoxicity of epirubicin on GCs

**Fig. 2:**
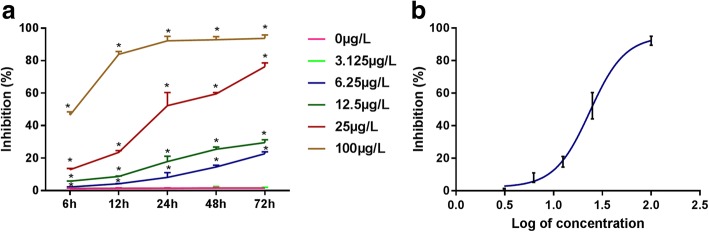
CCK8 assays of epirubicin-treated GCs. **a** The relationship between the inhibitory effect of epirubicin on GC proliferation and the concentration and duration of epirubicin. **b** The half-inhibitory concentration (IC50) 24 h after treatment was 25 μg/L. *P < 0.05

CCK-8 assay was performed to determine the cytotoxicity of epirubicin on human ovarian GCs. The administration of 6.25, 25, and 100 μg/L epirubicin significantly inhibited GC proliferation compared with that in the control group (*P* < 0.05), whereas that of 3.125 μg/L epirubicin showed no significant inhibitory effect on GC proliferation (*P* > 0.05). The inhibitory effect was positively correlated with the concentration and duration of epirubicin (Fig. 2a). Thus, the duration was set to 24 h, and the corresponding half-inhibitory concentration (IC50) was 25 μg/L (Fig. 2b).

### MB-MSCs restored epirubicin-induced hormone reduction of GCs

**Fig. 3:**
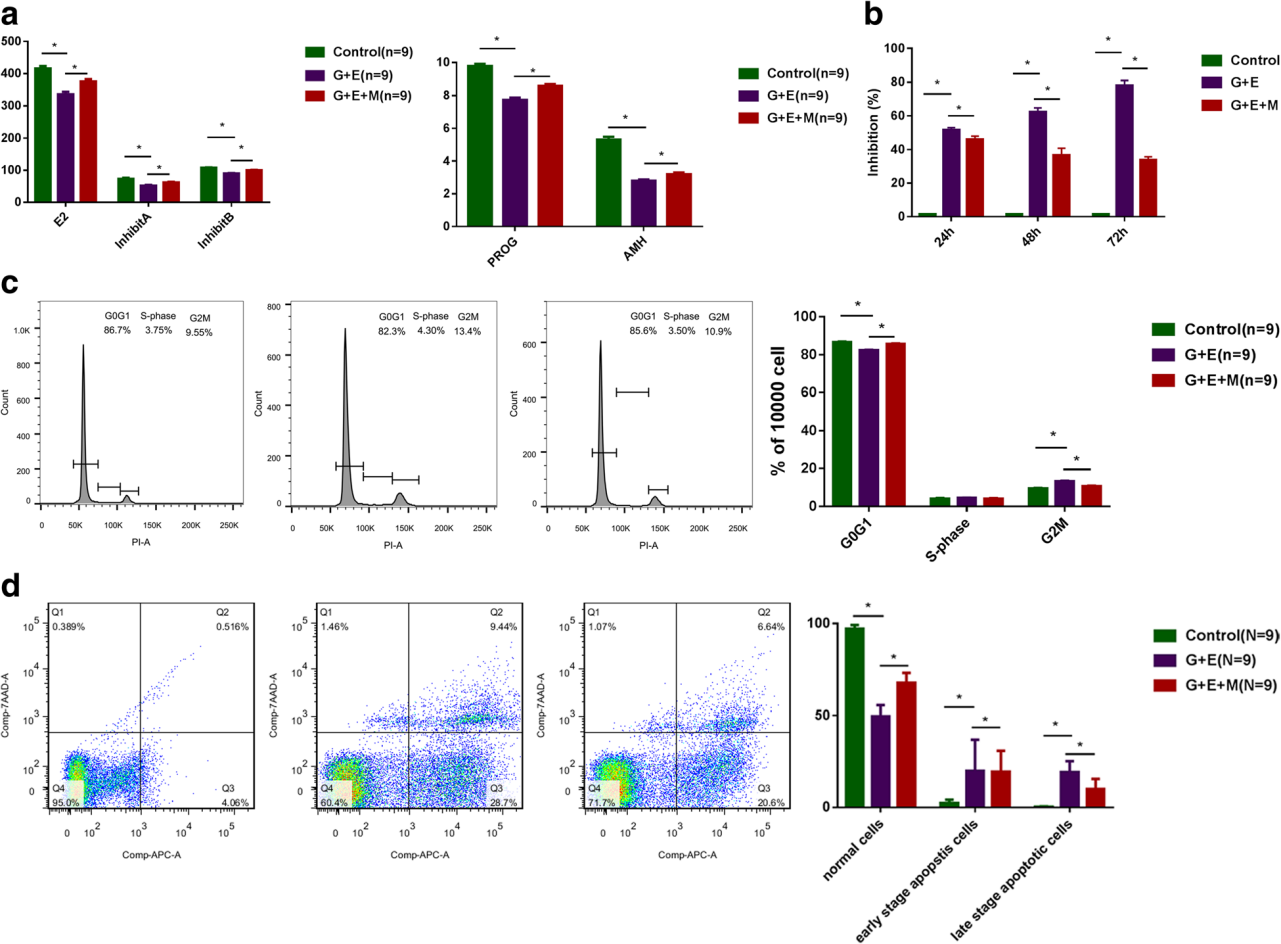
The effect of MB-MSCs on epirubicin-induced GC damage. **a** Hormone changes of GCs with different treatments. **b** The effect of MB-MSCs on epirubicin-induced inhibition of GC proliferation was detected with CCK8 arrays. **c** The effect of MB-MSCs on epirubicin-induced cell cycle arrest of GCs was analyzed with FACS. **d** Apoptosis of GCs with different treatments by flow cytometry analysis. *P < 0.05

The ELISA analysis showed that epirubicin reduced the secretion of hormones (E2, progesterone, AMH, inhibin A, and inhibin B) by human ovarian GCs (*P* < 0.05), which however could be restored to a certain extent by MB-MSCs (*P* < 0.05) (Fig. 3a).

### The effect of MB-MSCs on epirubicin-induced inhibition of GC proliferation

CCK8 assays were performed to examine GC proliferation. Compared with the control group, epirubicin inhibited GC proliferation in the G+E group (*P* < 0.05), which however was reversed by MB-MSCs in the G+E+M group (*P* < 0.05) (Fig. 3b).

### The effect of MB-MSCs on epirubicin-induced cell cycle arrest of GCs

FACS was performed to examine the effect of MB-MSCs on epirubicin-induced cell cycle of GCs (Fig. 3c). Compared with the control group, the ratio of G2/M phase was increased, while that of G1 phase was decreased in the G+E group (*P* < 0.05). However, compared with the G+E group, the ratio of G2/M phase was decreased, while that of G1 phase was increased in the G+E+M group (*P* < 0.05).

### MB-MSCs recovered epirubicin-induced apoptosis of GCs

The annexin V and 7-AAD double staining showed that the apoptosis of GCs cultured alone was significantly lower than that co-cultured with 25 μg/L of epirubicin for 24 h (4.58% vs 36.14%, respectively; *P* < 0.05). The early and late apoptotic percentages were 28.7% and 9.44% in the G+E group, which were significantly higher than those in the control group (4.06% and 0.51%, respectively, *P* < 0.05), but lower than those in the G+E+M group (19.54% and 9.99%, respectively, *P* < 0.05), indicating that MB-MSCs recovered epirubicin-induced apoptosis of human ovarian GCs (Fig. 3d).

### The effect of MB-MSCs on epirubicin-induced gene expressions of GCs

**Fig. 4:**
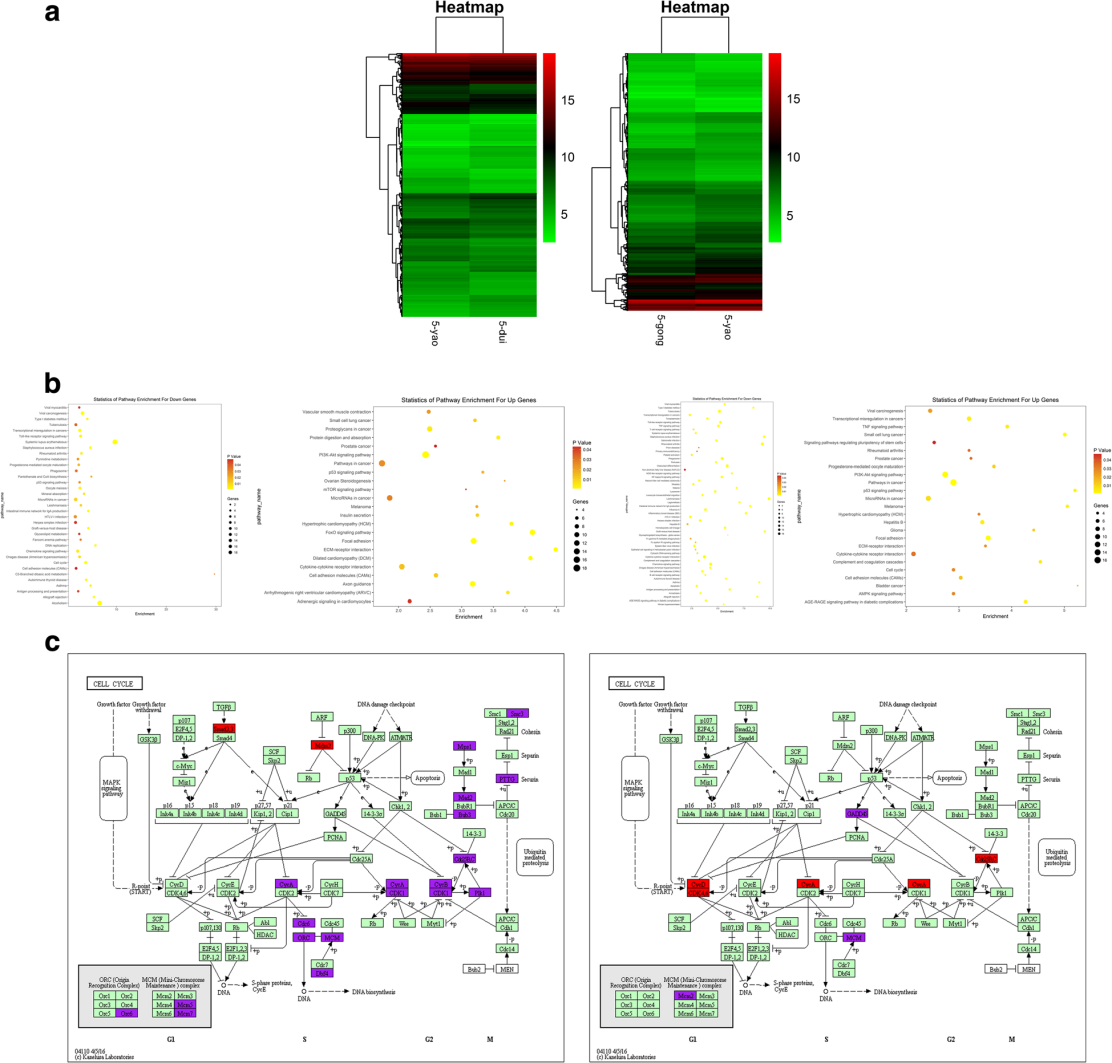
The effect of MB-MSCs on epirubicin-induced gene expressions of GCs. **a** The hierarchical cluster analysis of the mRNA level of GCs in different groups. **b** Enrichment analysis of differentially expressed gene pathways in GCs in different groups. **c** Differential gene mapping graph in cell cycle pathway of GCs in different groups

We found that MB-MSCs could repair epirubicin-induced GC damage, which might be related to cell proliferation, cell cycle, and cell apoptosis. A genome-wide transcriptional analysis using the Affymetrix GeneChip® identified 3599 significantly differentially expressed genes between G+E and control groups, including 1962 upregulated genes and 1607 downregulated genes, and 2814 significantly differentially expressed genes between G+E+M and G+E groups, including 1130 upregulated genes and 1684 downregulated genes (Fig. 4a). The pathway analysis using the KEGG database showed that the cell cycle pathway was involved in 18 differentially expressed genes between G+E and control groups and 7 differentially expressed genes between G+E+M and G+E groups (Fig. 4b), including CyclinB1 (fold change> 1.5, *P* < 0.05), CDC2 (fold change> 1.5, *P* < 0.05), and Gadd45b (fold change> 1.5, *P* < 0.05) (Fig. 4c). The microarray analysis results showed that of the three subtypes (Gadd45a, Gadd45b, and Gadd45g) of Gadd45, Gadd45b was identified as a significantly differentially expressed gene.

### The effect of MB-MSCs on epirubicin-induced protein expressions of GCs

**Fig. 5:**
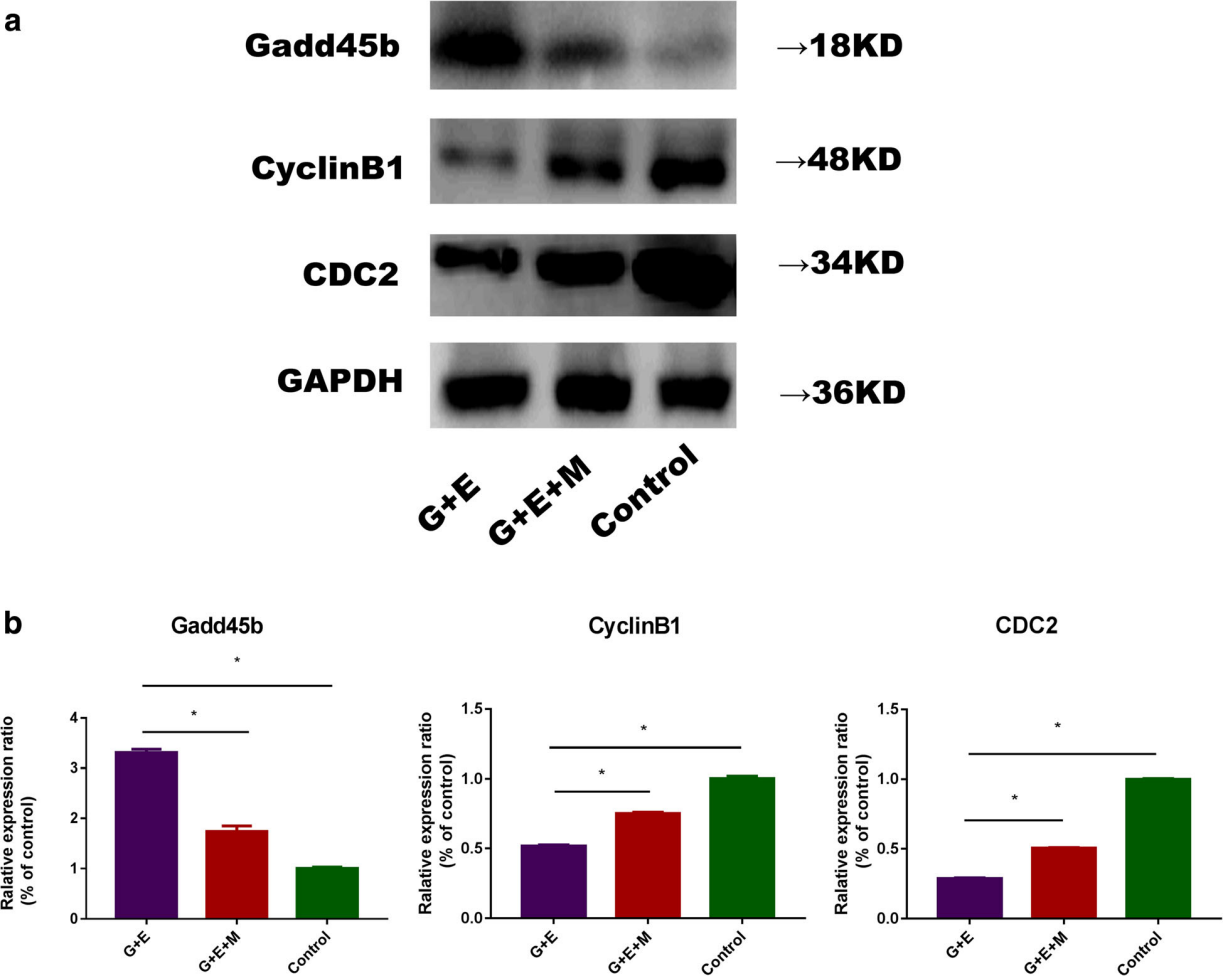
The effect of MB-MSCs on epirubicin-induced GC protein expressions: **a** Gadd45b, CyclinB1, and CDC2 protein expressions were analyzed with Western blot analysis. GAPDH was used as an endogenous control. **b** The quantitative graph of Gadd45b, CyclinB1, and CDC2 protein expressions. *P < 0.05

Western blot analysis showed that compared with the control group, CyclinB1 and CDC2 were downregulated (*P* < 0.05) and Gadd45b was upregulated (*P* < 0.05) in the G+E group. However, compared with the G+E group, CyclinB1 and CDC2 were upregulated (*P* < 0.05) and Gadd45b was downregulated (*P* < 0.05) in the G+E+M group (Fig. 5a, b).

## Discussion

Long-term administration of high-dose anthracycline can have a detrimental effect on the ovarian functions of young patients, resulting in amenorrhea, infertility, and even POF [16]. Bar-Joseph et al. [17] found that follicular germinal vesicle oocytes were more vulnerable to the toxic effect of doxorubicin than ovulated MII oocytes in mice. Ben et al. [18] found that in vivo administration of doxorubicin resulted in a significant reduction in the population of secondary follicles and a nearly significant reduction in the population of primordial and primary follicles in female mice. There is also accumulating evidence that adriamycin can induce GC apoptosis and follicle damage [6, 7]. GCs can provide essential nutrients for oocyte development and follicle maturation, and they are also implicated in mediating the regulation of gonadotropin on oocyte maturation and help to maintain a supportive microenvironment for oocyte maturation by autocrine and paracrine [19]. Thus, the apoptosis of GCs can directly affect follicle development, resulting in a reduction in ovulation rates and hormone levels and even the occurrence of POF. In this study, we investigated whether MB-MSCs could repair epirubicin-induced cytotoxicity in human ovarian GCs, and the results may contribute to developing more effective treatment strategies for chemotherapy-induced POF.

In this study, ovarian GCs were obtained from human follicular fluid using density gradient centrifugation, and these GCs were dendritic or spindle shaped with uniform and abundant cytoplasmic particles. IHC staining showed that FSHR, the specific marker of GCs, was expressed in both cell membrane and cytoplasm. In order to investigate whether MB-MSCs could repair epirubicin-induced damage to ovarian GCs, we isolated and characterized MB-MSCs as previously described [15], and co-cultured GCs treated by epirubicin with MB-MSCs.

Ovarian reserve is established during fetal life and depends critically on the number of primordial follicles in the ovarian cortex. However, the initial stock of primordial follicles decreases with chronological age until menopause, the final step of ovarian aging [20]. It is very important to evaluate the ovarian reserve as it is a good indicator of the reproductive potential and lifespan. However, it cannot be quantified directly in vivo or indirectly by menstrual status, and thus, hormonal and ultrasonographic assays have been developed for the detection of indirect markers, including E2, inhibin B, and AMH [21]. Among these markers, serum AMH has been shown to be the most accurate marker in estimating the pool of antral follicles, which can indirectly reflect the remaining ovarian reserve and the reproductive lifespan [22, 23].

In this study, the E2, progesterone, AMH, inhibin A, and inhibin B levels were determined to evaluate the functions of GCs. The ELISA results showed that epirubicin could inhibit the secretion of E2, progesterone, AMH, inhibin A, and inhibin B by GCs, indicating that the functions of GCs could be affected by epirubicin. However, compared with the G+E group, the E2, progesterone, AMH, inhibin A, and inhibin B levels were increased in the G+E+M group, indicating that MB-MSCs had the potential to recover epirubicin-induced cell damage.

CCK8 assays were performed to examine GC proliferation, and FACS was performed to determine the effect of MB-MSCs on epirubicin-induced cell cycle distribution of GCs. It was found that epirubicin inhibited GC proliferation and arrested them in G2/M phase, while MB-MSCs promoted GC proliferation and repaired epirubicin-induced G2/M phase arrest of GCs. Cell growth can be restrained by controlling the cell cycle, and the cell division cycle is regulated by the Cdk family of serine/threonine kinases and cyclins. G2/M transition is regulated by CyclinB1/CDC2 complex in mammalian cells [24, 25], whose activity is in turn regulated by CDC25C [26], p53 [27, 28], p21 [29], 14-3-3 s [30], and Gadd45 (growth arrest and DNA damage inducible gene) [31, 32]. To further investigate whether CyclinB1, CDC2, and their upstream regulatory factors were involved in the repair effect of MB-MSCs, we performed a genome-wide transcriptional analysis using the Affymetrix GeneChip®. It was found that 18 differentially expressed genes between G+E and control groups and 7 differentially expressed genes between G+E+M and G+E groups were involved in cell cycle pathway (Fig. 4c), including CyclinB1 (fold change> 1.5, *P* < 0.05), CDC2 (fold change> 1.5, *P* < 0.05), and Gadd45b (fold change> 1.5, *P* < 0.05). Thus, Gadd45b may play a vital role for MB-MSCs in repairing epirubicin-induced cell cycle arrest and promoting cell proliferation, and it can also act on the CyclinB1/CDC2 complex and thus affect GC cycle and proliferation.

The Gadd45 family includes Gadd45a, Gadd45b, and Gadd45g. Gadd45 genes encode small (18 kDa) evolutionarily conserved proteins that are highly homologous to each other (55–57% overall identity at the amino acid level), and they are highly acidic and localized in both cell cytoplasm and nucleus [33–35]. Gadd45 proteins have been implicated in stress signaling in response to physiological and environmental stressors, including oncogenic stressors, which can result in cell cycle arrest, DNA repair, cell survival, senescence, and apoptosis. The function of Gadd45 as a stress sensor is mediated via physical interactions with other cellular proteins implicated in cell cycle regulation and the response of cells to stress, notably PCNA, p21, CDC2/CyclinB1, p38, and JNK stress response kinases [36]. The inhibition of the endogenous expression of Gadd45a, Gadd45b, or Gadd45g in human cells by antisense Gadd45 constructs was found to impair the G2/M checkpoint following exposure to UV radiation or MMS [35, 37, 38]. Microinjecting a Gadd45a expression vector into primary human fibroblasts arrested cells at the G2/M boundary of the cell cycle [38], which could be attributed to their ability to inhibit the kinase activity of the CDC2/CyclinB1 complex [35, 39]. The association of Gadd45a/Gadd45b proteins with CDC2/CyclinB1 results in the dissociation of the CDC2/CyclinB1 complex, which in turn can inhibit CDC2 kinase activity [35]. Western blot analysis showed that epirubicin induced the expression of Gadd45b proteins and inhibited the expression of CyclinB1 and CDC2 proteins in GCs, while MB-MSCs inhibited the expression of Gadd45b proteins and induced the expression of CyclinB1 and CDC2 proteins in epirubicin-treated GCs. It could be inferred that epirubicin inhibited the activity of CyclinB1/CDC2 complex by inducing the expression of Gadd45b, resulting in G2/M phase arrest of GCs and inhibition of GC proliferation, while MB-MSCs promoted the transformation of GCs from G2 to M phase and GC proliferation by inhibiting the expression of Gadd45b in epirubicin-treated GCs.

Gadd45 proteins have a pro-apoptotic function. For example, blocking Gadd45b by antisense expression in M1 myeloblastic leukemia cells impaired TGFb-induced cell death, and thus, Gadd45b may be a positive modulator of TGFb-induced apoptosis [40]. In this study, we observed an increase in the apoptosis of GCs after epirubicin treatment and the expression of Gadd45b, which however could be inhibited by MB-MSCs. Therefore, the inhibitory effect of MB-MSCs on epirubicin-induced GC apoptosis might be related to the decreased expression of Gadd45b.

It was found that epirubicin might inhibit the activity of CyclinB1/CDC2 complex by inducing the expression of Gadd45b, resulting in the inhibition of GC proliferation and G2/M phase arrest of GCs, while MB-MSCs promoted GC proliferation, which might be related to the inhibition of the expression of Gadd45b, resulting in the inhibition of CyclinB1/CDC2 complex activity and the transformation of GCs from G2 to M phase. It was also observed that epirubicin might induce the apoptosis of GCs by inducing the expression of Gadd45b, while MB-MSCs might inhibit the apoptosis of GCs by inhibiting the expression of Gadd45b.

## Conclusions

MB-MSCs improved the secretion of E2, progesterone, AMH, inhibin A, and inhibin B; inhibited epirubicin-induced G2/M phase arrest of GCs; promoted GC proliferation; and inhibited GC apoptosis. The repair effect of MB-MSCs might be related to the inhibition of Gadd45b protein expression. These results indicate the possibility of MB-MSCs as a potential therapeutic strategy for the treatment of epirubicin-induced POF. This study also provides an experimental basis for the pathological mechanism and hormone replacement therapy for POF.

## Abbreviations

AMH: Anti-Müllerian hormone
CCK8: Cell Counting Kit-8 assays
DMEM/F12: Dulbecco’s modified Eagle’s medium
E2: Estradiol
ELISA: Enzyme-linked immunosorbent assay
FSHR: Follicle-stimulating hormone receptors
Gadd45: Growth arrest and DNA damage inducible gene
GCs: Granulosa cells
GnRH: Gonadotropin-releasing hormones
HCG: Human chorionic gonadotropin
HRT: Hormone replacement therapy
IHC: Immunohistochemical
IVF-ET: In vitro fertilization-embryo transfer
KEGG: Kyoto Encyclopedia of Genes and Genomes
MB-MSCs: Mesenchymal stem cells isolated from menstrual blood
MSCs: Mesenchymal stem cells
PBS: Phosphate-buffered saline
POF: Premature ovarian failure
